# Muscle Atrophy Due to Nerve Damage Is Accompanied by Elevated Myofibrillar Protein Synthesis Rates

**DOI:** 10.3389/fphys.2018.01220

**Published:** 2018-08-31

**Authors:** Henning T. Langer, Joan M. G. Senden, Annemie P. Gijsen, Stefan Kempa, Luc J. C. van Loon, Simone Spuler

**Affiliations:** ^1^Experimental and Clinical Research Center, a Joint Cooperation of Max Delbrück Center for Molecular Medicine and Charité – Universitätsmedizin Berlin, Berlin, Germany; ^2^Berlin-Brandenburg Center for Regenerative Therapies, Charité – Universitätsmedizin Berlin, Berlin, Germany; ^3^Charité – Universitätsmedizin Berlin, Berlin, Germany; ^4^Department of Human Biology, NUTRIM School of Nutrition and Translational Research in Metabolism, Maastricht University Medical Centre+, Maastricht, Netherlands; ^5^Berlin Institute of Health, Berlin, Germany; ^6^Max Delbrück Center for Molecular Medicine in the Helmholtz Association, Berlin, Germany

**Keywords:** skeletal muscle, atrophy, muscle loss, myofibrillar, protein synthesis, nerve damage, stable isotope, deuterium oxide

## Abstract

Muscle loss is a severe complication of many medical conditions such as cancer, cardiac failure, muscular dystrophies, and nerve damage. The contribution of myofibrillar protein synthesis (MPS) to the loss of muscle mass after nerve damage is not clear. Using deuterium oxide (D_2_O) labeling, we demonstrate that MPS is significantly increased in rat *m.* *tibialis anterior* (TA) compared to control (3.23 ± 0.72 [damaged] to 2.09 ± 0.26%^∗^day^−1^ [control]) after 4 weeks of nerve constriction injury. This is the case despite substantial loss of mass of the TA (350 ± 96 mg [damaged] to 946 ± 361 mg [control]). We also show that expression of regulatory proteins involved with MPS (p70s6k1: 2.4 ± 0.3 AU [damaged] to 1.8 ± 0.2 AU [control]) and muscle protein breakdown (MPB) (MAFbx: 5.3 ± 1.2 AU [damaged] to 1.4 ± 0.4 AU [control]) are increased in nerve damaged muscle. Furthermore, the expression of p70s6k1 correlates with MPS rates (*r*^2^ = 0.57). In conclusion, this study shows that severe muscle wasting following nerve damage is accompanied by increased as opposed to decreased MPS.

## Introduction

Skeletal muscle is the biggest organ of the human body, comprising at least 40% of its mass and containing 50–75% of all body proteins (Frontera and Ochala, 2015). It is pivotal to health and locomotion, and the lack of muscle mass and strength is associated with severely reduced independence, quality of life, and life expectancy (Metter et al., 2002; Wannamethee et al., 2007). Many clinical conditions are accompanied by muscle loss, such as cancer, COPD, or heart failure (Rosenberg, 1997; Al-Majid and McCarthy, 2001; Marquis et al., 2002; Thomas, 2007). Currently, no drug treatment for muscle wasting is available, with exercise and ample protein intake being the only *bona fide* intervention to slow muscle loss (Sepulveda et al., 2015; Garber, 2016). However, there are situations of muscle loss where physical activity is not an option. Such as in patients with fractures, critically ill patients or nerve damage. Peripheral nerve damage is a frequently occurring clinical condition that can be caused by disease or trauma (Dyck, 2005). A common model to study peripheral nerve damage is chronic constriction injury to the nerve (Bennett and Xie, 1988). Chronic constriction injury to the nerve is accompanied by debilitating symptoms such as neuropathic pain, hampered motor function and skeletal muscle atrophy (Bennett and Xie, 1988). Even though nerve function may recover, this is often outlasted by the deteriorating effects on muscle tissue. Although chronic nerve constriction is well investigated in respect to its implications for pain in animals, very little is known on the physiology of muscle wasting.

Muscle mass is determined by the balance between muscle protein synthesis (MPS) and muscle protein breakdown (MPB). Either side of the balance may be disturbed. Consequently, most muscle atrophies are assumed to show a combination of decreased MPS and increased MPB (McKinnell and Rudnicki, 2004). Yet, the individual contribution of decreased MPS may differ between various types of atrophies. For example in disuse atrophy in humans, blunted MPS rates seem to be the predominant cause for a decline in muscle mass (Wall et al., 2013a,b, 2016). Similarly, decreased MPS has been reported for starvation, sarcopenia, cachexia, and other conditions of muscle wasting, indicating a potential benefit of interventions which increase MPS (Emery et al., 1984; Yarasheski et al., 1993; Hector et al., 2018). In nerve damage induced atrophy, early work has suggested varying effects of denervation on MPS. Depending on the time point, decreased as well as transiently increased MPS rates were found in very young rats after nerve transection (Goldspink, 1976, 1978; Goldspink et al., 1983). How MPS rates are affected by chronic nerve constriction in adult animals is not known. Furthermore, previous studies using stable isotope labeling with the flooding dose- or primed continuous infusion technique were restricted to an MPS assessment period of a few hours, reducing their ability to predict absolute changes in muscle mass (Mitchell et al., 2014; Reid et al., 2014). The reemergence of deuterium oxide (D_2_O) as a mean to study integrated MPS *in vivo* over multiple days- or weeks, however, offers an attractive solution for this problem (Busch et al., 2006; Wilkinson et al., 2014; Damas et al., 2016).

Therefore we set out to investigate the effects of chronic nerve constriction on MPS. We combined long term D_2_O-mediated tracer experiments in adult rats with absolute changes in muscle mass, immunohistochemical analysis and protein expression data. We hypothesized that nerve constriction would cause a decrease in MPS rates. However, we found that despite substantial muscle loss, nerve damage induced atrophy is accompanied by chronically elevated as opposed to reduced myofibrillar protein synthesis (MPS) rates.

## Materials and Methods

### Ethical Approval and Animal Experiments

The animal experiments were approved by the local authority (Landesamt für Gesundheit und Soziales, Berlin, Germany) under the reference G 0083/15 and performed at the animal care unit of the Max Delbrück Center for Molecular Medicine (MDC, Berlin).

### Nerve-Damage Model

Ten male Sprague-Dawley rats [Crl:CD (SD), Charles River, Sulzfeld, Germany] between 20–21 weeks of age were housed in individual cages. The animals were fed a diet of 20 g chow (ssniff Spezialdiäten GmbH, Soest, Germany) (**Supplementary Figure 1**) equivalent to 79 kcal^∗^day^−1^ to slow down commonly occurring weight gain. Nerve damage was induced via chronic constriction injury to the sciatic nerve (Sommer, 2013). The rats were anesthetized via isoflurane inhalation (∼2.5%) and treated with an injection of 4–5 mg carprofen ^∗^kg^−1^ bodyweight to reduce postsurgical pain. An incision was made along the femur, and the *vastus lateralis* was disconnected from the *biceps femoris* by blunt dissection. The sciatic nerve was exposed above the point of trifurcation and constriction injury was induced by implanting a cuff around the nerve. To further reduce postsurgical pain of the animals, they received 100 mg ^∗^kg^−1^ metamizole. For the last 2 weeks, the animals were electrostimulated twice a week to maintain the nerve injury and impair recovery, as has been described previously (Baptista et al., 2008; Gigo-Benato et al., 2010). The animals were dissected 4 weeks post surgery, between the age of 24 and 25 weeks. In a fasted state, the animals were put under deep anesthesia via isoflurane inhalation (∼3.5%) and the TA and *extensor digitorum longus* (EDL) were collected. Muscles were quickly weighed and cut in half, with one part being immediately snap frozen in liquid nitrogen and the other part being embedded in gum tragacanth for histological analysis and frozen in isopentane.

### D_2_O Labeling Protocol

We used a labeling protocol suitable to detect deuterium (^2^H) enrichments in alanine of the myofibrillar protein fraction of skeletal muscle via GC-MS similar to what has been published previously (Busch et al., 2006; Gasier et al., 2009). Briefly, 2 weeks after surgery the animals received an intraperitoneal injection of 0.014 mL ^∗^g^−1^ bodyweight of D_2_O (99.8%+ Atom D, Euriso-Top GmbH Saarbrücken) and 0.9% NaCl. This injection primed the animals and enriched their body water levels to approximately 2.5% D_2_O. To maintain the label concentration, the rats received drinking water with 4% ^2^H_2_O enrichment.

### Myofibrillar Protein Extraction

Myofibrillar protein isolation was performed as described previously (Burd et al., 2012). Briefly, 80–120 mg muscle sample of rat TA (*n* = 10) was weighed into an Eppendorf tube and stored on ice. A standard buffer solution was added to each sample at 10 μL ^∗^mg^−1^ and the muscle tissue was thoroughly homogenized. Scissors were used to mince the tissue before subsequent homogenization by plastic pestles. To fractionate a pellet rich in myofibrillar- and other structural proteins, the sample was spun at 700 *g* for 10 min at 4°C. The remaining pellet was washed twice with buffer and dH_2_O, the supernatant was discarded and 1 mL 0.3 NaOH was added to the pellet to further solubilize the myofibrillar proteins and isolate them from collagen. The samples were heated at 50°C for 30 min. Subsequently the sample was spun at 10,000 *g* for 5 min at 4°C and the supernatant containing the myofibrillar protein was transferred into 4 mL screw-cap glass vials. One milliliter of 1M PCA was added to each glass vial to denaturate the remaining proteins. After centrifugation, the supernatant was removed and the pellet washed twice with 500 μL 70% EtOH. After removal of the EtOH, 1.5 mL of 6M HCL was added to hydrolyze the samples over night at 110°C. The next day the samples were put in a heating block (120°C) and dried under a nitrogen steam. To further purify the amino acids, the samples were passed through Dowex exchange resin (AG 50W-X8 Resin, Bio-Rad) prior to derivatization. After purification, the glass vials were carefully vortexed and put under a nitrogen steam to dry before derivatization. Samples containing the free amino acids of the myofibrillar protein fraction were then converted to their tert-butyldimethylsilyl (TBDMS) derivatives via the addition of 50 μL of N-tert-Butyldimethylsilyl-N-methyltrifluoroacetamide (MTBSTFA) and 50 μL of acetonitrile to the sample. Each sample was then incubated for 1 h at 70°C. The sample was then transferred to 2 mL screw-cap chromacol vials (Thermo Fisher Scientific, Schwerte, Germany) suitable for GC-MS injection.

### Plasma Protein Extraction

To precipitate plasma protein, 40 μL perchloric acid (20%) were added to 360 μL plasma sample. After vortexing, free amino acids were separated from protein bound amino acids by centrifugation (3500 rpm, 20 min, 4°C). The pellet was collected and washed three times with 1 mL perchloric acid (2%) before being hydrolyzed over night as described above. After hydrolysis, samples were purified and processed for GC-MS injection as described above. Values of unlabeled samples were used as a baseline control for ^2^H enrichment in plasma protein bound alanine.

### Free Alanine Enrichments in Plasma

Plasma samples were thawed on ice and dry 5-sulfosalicylic acid was added to the sample to deproteinize it as described previously (Trommelen et al., 2016). After vortexing, the sample was spun at 1000 *g* for 15 min. The supernatant was collected and then purified, processed and measured on the GC-MS as described in the sections above.

### GCMS Measurement and Stable Isotope Enrichment Analysis

The alanine enrichment was determined by electron ionization gas chromatography-mass spectrometry (GC-MS; Agilent 6890N GC/5973N MSD) using selected ion monitoring of masses 232, 233, 234, 235, and 236 for their unlabeled and labeled H2-alanine. We applied standard regression curves to assess linearity of the mass spectrometer and to control for the loss of tracer.

### Immunoblotting

Approximately 400 μm of sample was cut from the histology block and homogenized in a standard lysing buffer using a pestle. Protein concentrations in the samples were determined using a bicinchoninic acid assay (BCA) kit (Thermo Fisher Scientific, Schwerte, Germany). The required volume for 40 μg of protein per sample was calculated, aliquoted and SDSPP (6×) and SDSPP in H_2_O (1×) were added for a total volume of 15 μL. Commercial SDS gels (Invitrogen NuPAGE Bis-Tris Gel, Thermo Fisher Scientific, Schwerte, Germany) and electrophoresis (130–200 V) were used to separate the proteins in each sample. The semi-dry blot technique was used for transfer (45 min at 18 V). Membranes were blocked in TBS-T (4% milk powder) for 1 h at room temperature. Membranes were then incubated with the first antibody in TBS-T (4% milk powder) or BSA overnight (**Supplementary Figure 9**). The next day, samples were washed in TBS-T and the second antibody was added for 60 min at room temperature. After washing, chemiluminescence (ECL) was used for development of the bands. Expression levels of the protein bands of interest were directly analyzed using Image Studio Lite (LI-Cor, Lincoln, NE, United States). Protein loading and transfer was controlled for with a ponceau s staining (**Supplementary Figure 2**).

### Histochemistry and Immunofluorescence

Gomori trichrome and toluidine blue ATPase stainings were performed according to an established protocol (Engel and Cunningham, 1963; Ogilvie and Feeback, 1990). For the fiber type distribution, as many fibers per slice were measured as clearly distinguishable by the toluidine blue staining. For type 2 fibers this was approximately 50 per slide, for the far less abundant type 1 fibers approximately 10.

For immunofluorescence staining, freshly cut cryosections were left 1 h at RT to dry and then fixated in 3.7% paraformaldehyde. Subsequently, sections were washed and blocked in 3% BSA/PBS. Afterward sections were incubated with anti-GLUT4 antibody (**Supplementary Figure 9**), CT-3,-3/5; 1:1000 in PBS (1% BSA) for 1 h at room temperature. The sections were incubated with biotin anti rabbit (1:200) in PBS and Streptavidin-Cy3 (1:200). Nuclei were visualized with Hoechst (1:1000 in PBS) before being mounted on slides using Aqua Mount (Thermo Fisher Scientific, Schwerte, Germany).

Pictures were acquired using a Zeiss LSM 700 confocal microscope (Zeiss, Jena, Germany) and the associated vendor software Zen 2012. Mosaic pictures of the TA were created with a Leica DFC 420 microscope (Leica Microsystems, Wetzlar, Germany). Fiber number was analyzed by counting every single fiber of the section.

### Body Composition Analysis

Body composition was measured using the Minispec LF90 II time domain NMR analyzer (6.5 mHz, Bruker Optics, United States). Rats were placed into a restraint tube and inserted into the instrument which measured fat mass, fat-free mass, and fluid content of the animal.

### Fractional Synthesis Rate Calculations

Myofibrillar protein synthesis rates were calculated using the precursor-product method (Wall et al., 2013b).

$$\text{FSR }(\%*{\text{d}}^{-1})=(\Delta {\text{MPE}}_{\text{myo}}/(\Delta {\text{MPE}}_{\text{plasma}}*\text{t}))*100$$

where FSR is the fractional synthesis rate of myofibrillar proteins, ΔMPE_myo_ is the change in enrichment of ^2^H in muscle protein-bound alanine, ΔMPE_plasma_ is the change in enrichment of ^2^H in alanine found in plasma and t is time.

### Statistics

Statistical tests were applied as appropriate depending on the sample- and group number. Data are expressed as scatter dot plot with the line indicating the mean, mean ± standard deviation or floating bars (minimum to maximal value) with the line indicating the mean. After testing for normality of the data, Student’s *t*-test or ANOVA with Tukey’s *post hoc* test were applied depending on the number of groups. *p*-Values below 0.05 were deemed significant.

## Results

### Nerve Damage Induces Substantial Muscle Loss

**FIGURE 1:**
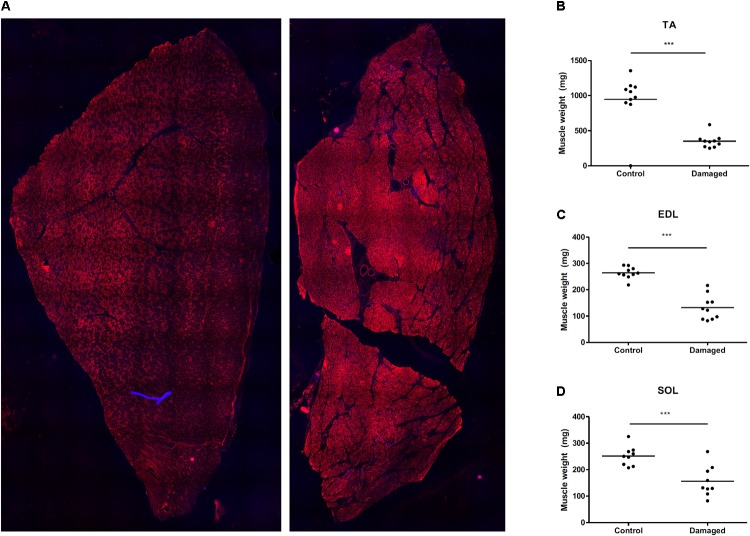
**(A)** Mosaic picture of rat *tibialis anterior* stained with GLUT4. **(B)** Nerve damage induces substantial muscle loss in *m. tibialis anterior* (TA) (*n* = 10), **(C)** *m. extensor digitorum longus* (EDL) (*n* = 10), and **(D)** *m. soleus* (SOL) (*n* = 9). ^∗^*p* < 0.05, ^∗∗^*p* < 0.01, ^∗∗∗^*p* < 0.001.

We induced sciatic nerve damage in otherwise healthy, male SD rats. We observed a fully developed muscle wasting phenotype at 28 days post surgery (**Figures 1A–D**). Immediately after surgery, the animals showed signs of decreased innervation of the hind limb affected, as expected after peripheral nerve injury (Gigo-Benato et al., 2010). The animals did not show any symptoms of decreased alertness or daily activity. Sciatic nerve innervated muscles lost significant mass: After 28 days TA mass had decreased by 66%, from 946 to 350 mg (**Figure 1B**), EDL by 50% from 264 to 132 mg (**Figure 1C**). In the *m. soleus* (SOL), loss of mass was less pronounced. Muscle weight decreased from 252 to 156 mg which approximates a 38% loss in muscle mass (**Figure 1D**). Since the SOL is almost exclusively composed of type 1 fibers (Gregory et al., 2001), this might hint toward a predominant type 2 fiber atrophy in association with disuse, rather than neurogenic atrophy. We followed this up via histological analysis.

### Fiber Atrophy and Deteriorated Body Composition

**FIGURE 2:**
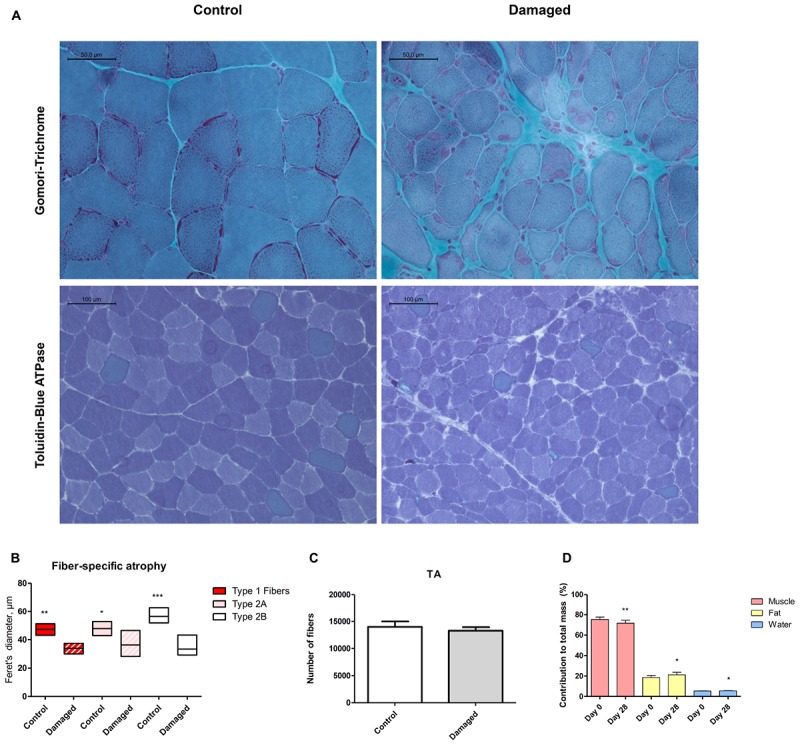
**(A)** Histochemical analysis via Gomori trichrome staining reveals regenerating fibers, centrally located nuclei and necrosis (upper panel). Toluidine blue staining shows fiber type specificity (lower panel). **(B)** Quantification of Feret’s diameter reveals pronounced type 2b fiber atrophy (*n* = 5). **(C)** No significant difference in number of muscle fibers between damaged and control muscle (*n* = 3). **(D)** Decreased lean body mass, increased fat mass and increased body water content 28 days post surgery (*n* = 8). ^∗^*p* < 0.05, ^∗∗^*p* < 0.01, ^∗∗∗^*p* < 0.001.

Histological analysis at day 28 after initiation of nerve damage revealed signs of necrotizing myopathy with regenerating fibers, fibers with centrally located nuclei, necrotic, and atrophic fibers (**Figure 2A**). The Feret’s diameter ranged from 43–51 μm in healthy type 1 fibers, and from 30–37 μm in damaged type 1 fibers (**Figure 2B**). In type 2a fibers, Feret’s diameter ranged from 43–53 μm in healthy muscle and 28–47 μm in damaged muscle (**Figure 2B**). Type 2b fibers ranged from 52–63 μm in healthy, and 29–43 μm in damaged muscle (**Figure 2B**). Overall, nerve damage induced a decrease in fiber diameter in all three fiber types (**Figure 2B**). In our study the type 2b fibers were most affected, decreasing by 41% (± 13%) in Feret’s diameter (**Figure 2B**). When type 2a and -b fibers were clustered, the loss of fiber diameter was greater than in type 1 fibers (**Supplementary Figure 3**). This confirms that the loss of muscle mass is predominantly based on type 2 fiber atrophy and explains why the SOL showed the least decline in muscle mass, being composed almost exclusively of type 1 fibers (Gregory et al., 2001). The total number of fibers in a complete cross section of TA from control and nerve damaged muscle were counted. In healthy muscle we found 13980 ± 999 compared to 13270 ± 652 fibers in damaged muscle (**Figure 2C**). These data indicate that muscular atrophy was due to loss of mass in individual fibers rather than reduction of total number of fibers, all consistent with muscular atrophy rather than dystrophy.

We asked whether sciatic nerve-induced atrophy resulted in overall changes in body composition. From day 0 to 28 days post surgery, we detected a 3.7% (± 1.3%) decrease in lean body mass from 75.4% (± 2.3%) to 71.7% (± 2.9%) (**Figure 2D**). The loss of lean body mass percentage occurred despite a tendency toward an increase in bodyweight (*p* = 0.61; **Supplementary Figure 7**). The decrease in lean body mass was accompanied by a slight increase in body fat percentage (**Figure 2D**).

### Increased Myofibrillar Protein Synthesis in Atrophic Muscle

**FIGURE 3:**
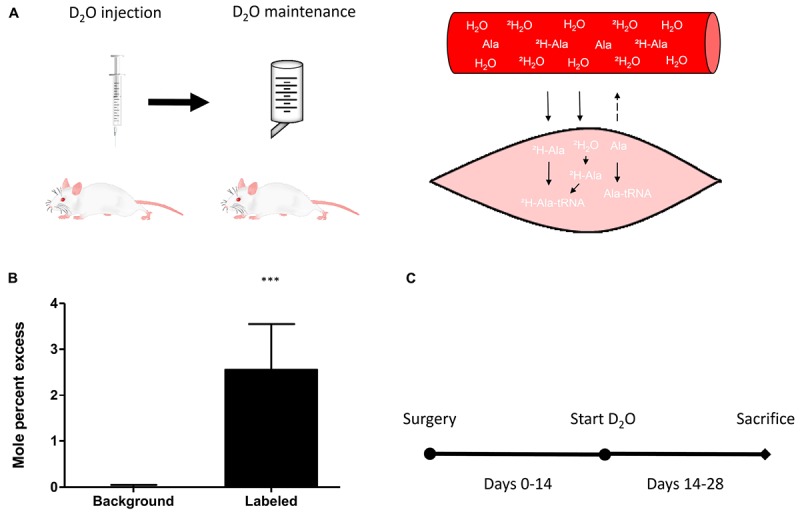
**(A)** Schematic principle of D_2_O labeling protocol in rats (left) and muscle (right). **(B)** Quantification of the incorporation of ^2^H into m+1 of the myofibrillar alanine fraction in unlabeled background muscle (*n* = 4) versus D_2_O labeled muscle (*n* = 40). **(C)** Schematic representation of the study protocol. Surgery at day 0, start of the labeling experiment with D_2_O labeling on day 14 and assessment of myofibrillar protein synthesis between day 14 and day 28. ^∗∗∗^*p* < 0.001.

**FIGURE 4:**
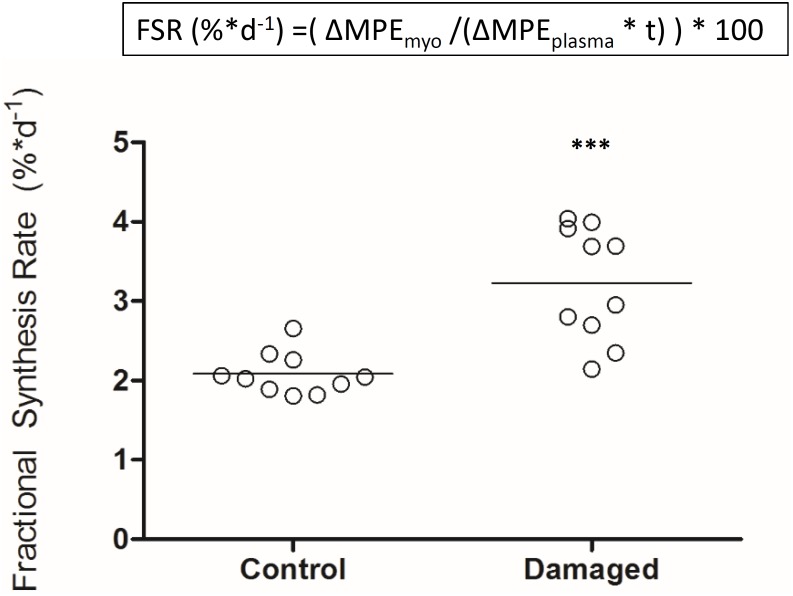
Myofibrillar fractional protein synthesis rates in nerve damaged TA and contralateral control (*n* = 10). ^∗∗∗^*p* < 0.001.

Deuterium oxide was injected and then added to regular water supply to analyze myofibrillar fractional synthetic rate (**Figure 3A**). We confirmed our ability to reliably detect ^2^H labeled alanine in a vast amount of different rat muscle samples, obtained from several interventions which have utilized D_2_O (**Figure 3B**). Myofibrillar fractional synthetic rate was increased 1.6-fold in the damaged compared to the control leg after 2 weeks of D_2_O treatment (3.23 ± 0.72 to 2.09 ± 0.26%^∗^day^−1^, respectively) (**Figure 4**). Every single animal (*n* = 10) showed increased muscle protein synthesis rates in the damaged compared to the control leg (**Supplementary Figure 4**). To our knowledge, this is the first study showing an integrated increase in MPS during a prolonged period of loss of muscle mass.

### Expression of Key Signaling Proteins Regulating Muscle Size

**FIGURE 5:**
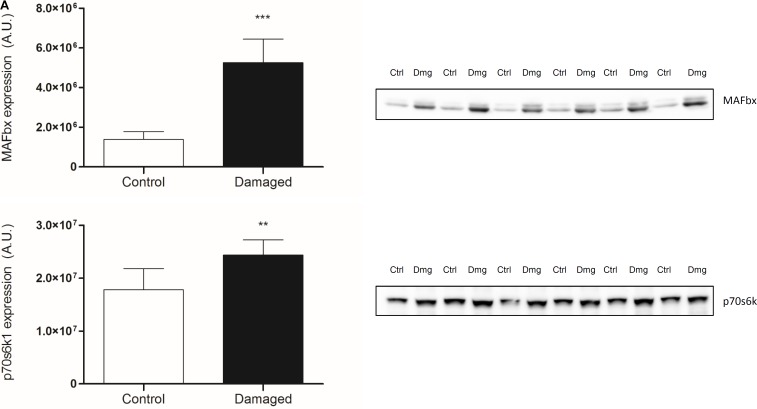
**(A)** Protein expression of MAFbx and p70s6k1 in contralateral (ctrl) and damaged (Dmg) rat TA; representative pictures of blots (*n* = 6). ^∗∗^*p* < 0.01, ^∗∗∗^*p* < 0.001.

**FIGURE 6:**
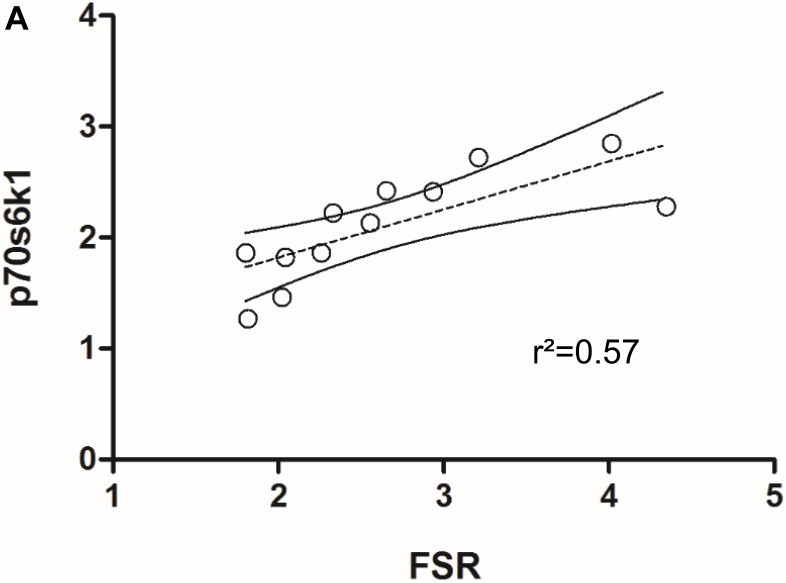
**(A)** Correlation between p70s6k1 protein expression and myofibrillar fractional synthesis rates (FSR) in rat TA (*n* = 6).

Skeletal muscle proteolysis is partially regulated by the E3 ubiquitine proteasome pathway and its muscle specific ligases MAFbx and MuRF1 (Bodine et al., 2001). To investigate how key signaling proteins of the proteasome pathway are regulated in our model, we investigated MAFbx and MuRF1 by Western blot analysis. Expression of MAFbx was increased fourfold in the damaged versus the control leg (5.3 ± 1.2 to 1.4 ± 0.4 AU, respectively) (**Figure 5A**, upper panel). MuRF1 expression followed a similar pattern (*p* < 0.0001) (**Supplementary Figure 5**). Protein expression of p70s6k1 increased 1.4-fold in the damaged leg (2.4 ± 0.3 to 1.8 ± 0.2 AU) (**Figure 5A**, lower panel). Phosphorylated p70s6k1 could not be detected (**Supplementary Figure 6**). We found a correlation between p70s6k1 expression and myofibrillar fractional synthesis rates (*r*^2^ = 0.57) (**Figure 6A**). The correlation for p70s6k1 and FSR is independent of the intervention effect and still present if the analysis is restricted to the control leg (*r*^2^ = 0.65) (**Supplementary Figure 8**).

## Discussion

The most prevalent assumption is that in most situations of muscle loss, there is a decrease in protein synthesis as well an increase in protein breakdown (McKinnell and Rudnicki, 2004). In disuse atrophy and immobilization in humans, a decrease in MPS appears to be the predominant mechanism causing muscle loss (Wall et al., 2013a; Phillips and McGlory, 2014). A decrease in MPS has also been observed for diet induced muscle atrophy in obese men, cancer cachexia, sepsis, and burned patients (Emery et al., 1984; Sakurai et al., 1995; Lang et al., 2007; Hector et al., 2018). In our study, we investigated chronic changes in MPS in response to nerve damage induced muscle atrophy. In stark contrast to the scenarios mentioned above, we found that MPS rates are increased as opposed to decreased during nerve damage induced muscle loss (**Figure 4**). Early studies on MPS after nerve damage have found varying results. MPS was reported to be transiently increased *in vitro* and *in vivo* by Buse, Goldspink and others (Buse et al., 1965; Goldspink, 1976, 1978). Later work has shown a decrease in MPS in muscle that has undergone compensatory growth with subsequent nerve transection (Goldspink et al., 1983). However, the implications of these studies differ profoundly from our findings. One reason are the differences between the nerve damage models: while a lot of work has been done on nerve transection, less is known for chronic constriction injuries to the nerve, which was the model in this study. In fact, no study thus far had investigated how muscle protein turnover and MPS are affected by nerve constriction injury. Furthermore, the studies finding an increase in tracer incorporation into the EDL and SOL after nerve transection were performed in particularly young rats (Goldspink, 1976, 1978). Undergoing age related growth, these animals still increased absolute mass of the denervated muscle over the course of the experiment (Goldspink, 1976, 1978). The result was slowed growth and relative atrophy of the affected muscles compared to control animals rather than absolute atrophy. In the face of a systemically anabolic environment, increased MPS rates may be less surprising. We chose full grown, adult rats (21–22 weeks old) and controlled their food intake to avoid excess bodyweight- and associated muscle gains (**Supplementary Figure 7**). Over the course of 4 weeks after the surgery our animals lost 66% of the TA compared to the contralateral control leg, and 50% of the EDL mass, respectively (**Figures 1B,C**). Despite this significant decrease in muscle mass we found a 1.6-fold increase in MPS in the TA (**Figure 4**). To our knowledge, this is the first study finding such a pronounced increase in integrated MPS despite absolute atrophy of the muscle. This supports the notion that MPS rates may be more indicative of muscle remodeling and ongoing regeneration than muscle growth *per se* (Ochala et al., 2011; Mitchell et al., 2014; Damas et al., 2016).

The timing to assess muscle protein turnover is crucial to the understanding of the changes in muscle mass. It is well known that the time course of muscle protein turnover in response to an atrophic stimulus is dynamic and depends on a variety of parameters. For example in muscle disuse atrophy, the majority of the changes to muscle protein turnover occur within the first week after the onset of the stimulus (Wall et al., 2016). It is thought that MPS decreases rapidly being accompanied by swift muscle loss, both tapering off in the second and third week of disuse (Wall et al., 2013a,b). Therefore, assessing muscle protein synthesis at a later time point may miss important changes. In respect to nerve damage, the literature suggests a fairly steady rate of muscle loss (Goldspink, 1976; al-Amood et al., 1991; Ma et al., 2007). It is important to note that the muscle continues to lose mass up to 3–12 months after nerve damage (Wu et al., 2014). To avoid any artifacts due to an initial inflammatory response induced by the surgery, we chose to analyze MPS during the second half of our intervention. We used D_2_O as a tracer to measure integrated protein synthesis over the course of 2 weeks (**Figure 3C**). As opposed to a short term experiment with the constant infusion- or flooding method, this allowed us to assess chronic alterations of MPS.

To investigate changes in protein expression that may be underlying the observed changes in protein turnover, we analyzed key signaling proteins for muscle protein synthesis and breakdown. For muscle protein synthesis we focused on p70s6k1, a protein downstream of mTORC1, known to increase protein synthesis upon phosphorylation and with a regulatory role in muscle growth (Baar and Esser, 1999; Saxton and Sabatini, 2017). To gain insight into the signaling underlying protein breakdown, we analyzed the E3 ubiquitine ligases MAFbx and MuRF1. These are muscle specific proteins downstream of FOXO, which have been shown to be upregulated under most atrophic conditions and are crucial regulators of muscle loss (Bodine et al., 2001; Gomes et al., 2001; Bodine and Baehr, 2014). In our model, protein expression of p70s6k1 is significantly increased in the nerve damaged leg compared to the control leg (**Figure 5A**, lower panel). The expression of p70s6k1 correlates with fractional synthesis rates of myofibrillar protein (**Figure 6A**). Interestingly that is still the case when expression and synthesis rates are analyzed exclusively in the control leg (**Supplementary Figure 8**). We tried to analyze phosphorylated p70s6k1, but failed to detect any in both, the damaged and the control legs. We confirmed the absence of phosphorylated p70s6k1 in our samples by the addition of positive controls (**Supplementary Figure 6**). The lack of phosphorylated p70s6k1 is not surprising, as the expression pattern appears to be transient and sampling of muscle would have to occur closely to the initiation of an early stimulus, which was not the case in our study (Ogasawara et al., 2013; West et al., 2016). Eventually, in case of our atrophy model the protein expression data seems to line up with the protein turnover data from the tracer experiments.

## Conclusion

In summary, we found that nerve damage induced muscle loss is primarily based on muscle fiber atrophy, not the loss of muscle fibers. With the combination of integrating the D_2_O tracer method with the analysis of absolute changes in muscle mass, we were able to find that in our model of muscle atrophy, muscle loss is accompanied by an increase as opposed to a decrease in MPS rates. These findings support the notion that muscle protein synthesis may be reflective of muscle remodeling and should not be used as a proxy to predict changes in muscle mass. In conclusion, muscle atrophy caused by chronic constriction injury to the nerve is not associated with a decline in MPS rates.

## Author Contributions

HL was responsible for the design of the study, animal experiments, analysis of the samples, interpretation of the data, and writing of the manuscript. JS, HL, and AG prepared the samples for GC-MS analysis. LvL was involved in the design of the study, interpretation of the data, and writing of the manuscript. SK was involved in the interpretation of the data. SS was involved in the design of the study, interpretation of the data, and writing of the manuscript.

## Conflict of Interest Statement

The authors declare that the research was conducted in the absence of any commercial or financial relationships that could be construed as a potential conflict of interest.
