# Supplementary material for: Muscle Atrophy Due to Nerve Damage Is Accompanied by Elevated Myofibrillar Protein Synthesis Rates
Source: Front Physiol. 2018 Aug 31;9:1220. doi: 10.3389/fphys.2018.01220 (PMC6127268; doi:10.3389/fphys.2018.01220)
Supplement: Supplementary file 1 [file Table_1.DOCX]

Supplementary Material

**Muscle atrophy due to nerve damage is accompanied by elevated myofibrillar protein synthesis rates**

Henning T. Langer^1,2,3*^, Joan M.G. Senden^6^, Annemie P. Gijsen^6^, Stefan Kempa^4,5^, Luc J.C. van Loon^6^, Simone Spuler^1-5^

***Corresponding author: Henning Langer, henning.langer@charite.de**

## Supplementary Figures

##
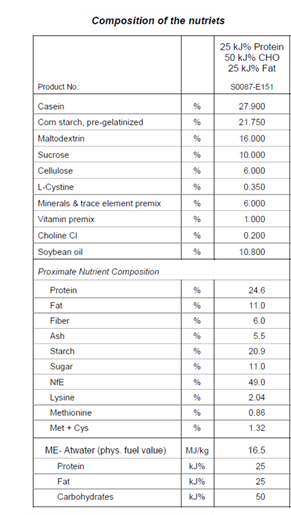


**Supplementary Figure 1.** Composition of the rat chow (ssniff Spezialdiäten GmbH, Soest, Germany).


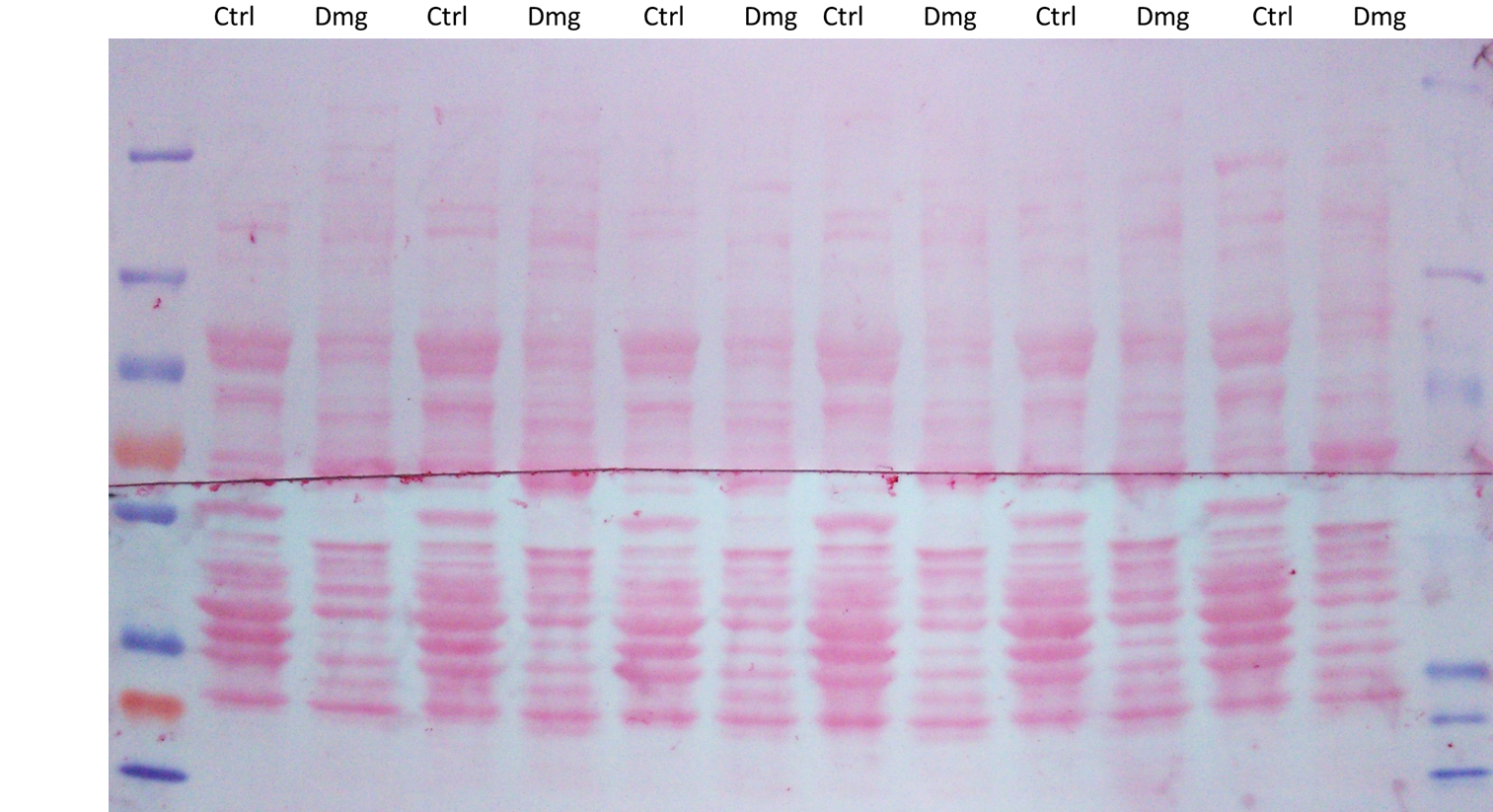


**Supplementary Figure 2.** Representative Ponceau S staining of a membrane which was used for western blot analysis.

**Supplementary Figure 3.** Clustered fiber type analysis. *N*=5 animals, counted were 10-50 fibers per slide depending on the quality of the Toluidine blue staining.

**Supplementary Figure 4.** Intraindividual comparison of myofibrillar protein synthesis rates of the TA in the contralateral control leg compared to the nerve damaged leg (*n*=10). The dashed lines connect the values of the contralateral TA (control) with the nerve damaged TA (damaged) of the same animal.

**
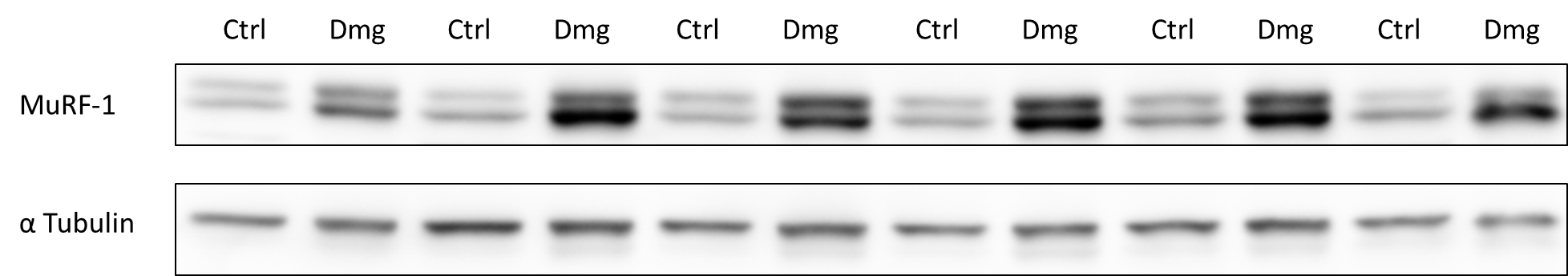
**

**Supplementary Figure 5.** Protein expression of MuRF-1 and Alpha Tubulin in the contralateral control TA (Ctrl) next to the nerve damaged TA (Dmg) of the same animal (*n*=6).


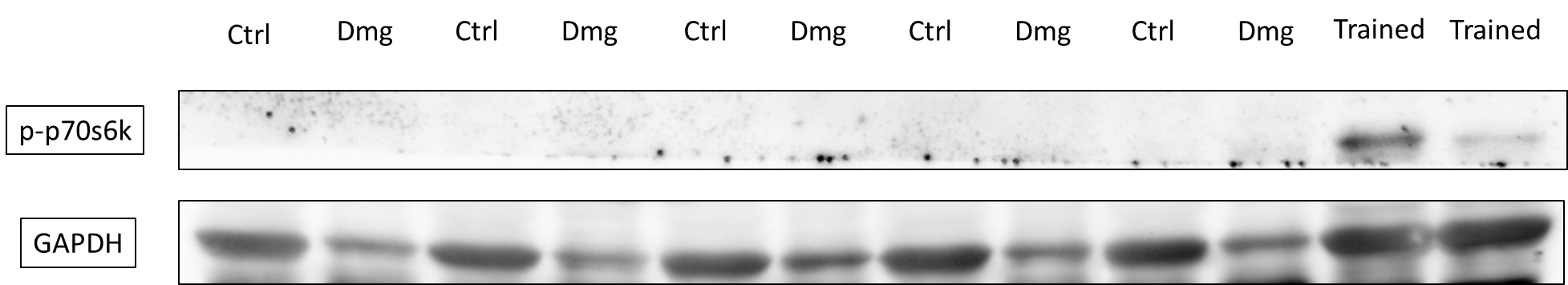


**Supplementary Figure 6.** Protein expression of phosphorylated p70s6k1 in the contralateral control TA (Ctrl) next to the nerve damaged TA (Dmg) of the same animal (*n*=5), as well as compared to a positive control (exercised rat TA (Trained)) (*n*=2).

**Supplementary Figure 7.** Change in bodyweight over the course of the 4 week experiment (*n*=8).

**Supplementary Figure 8.** Correlation between myofibrillar protein synthesis (FSR) and p70s6k1 expression under baseline conditions. Only within the contralateral control TA (*n*=6).


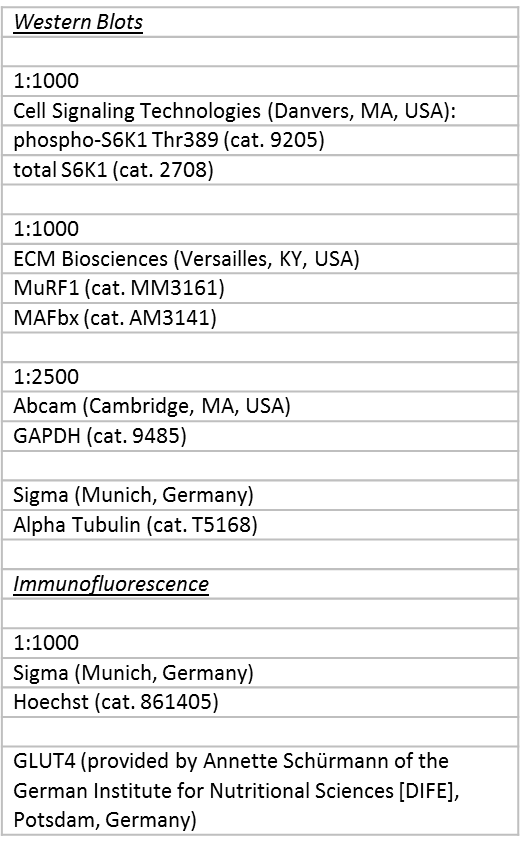


**Supplementary Figure 9.** List of antibodies and dilutions.
